# Nutritional value of high fiber co-products from the copra, palm kernel, and rice industries in diets fed to pigs

**DOI:** 10.1186/s40104-015-0056-6

**Published:** 2015-12-23

**Authors:** Hans Henrik Stein, Gloria Amparo Casas, Jerubella Jerusalem Abelilla, Yanhong Liu, Rommel Casilda Sulabo

**Affiliations:** Department of Animal Science, University of Illinois, Urbana, 61801 USA; Departamento de Producción Animal, Facultad de Medicina Veterinaria y de Zootecnia, Universidad Nacional de Colombia, Bogotá, Colombia; University of California, Davis, 95616 USA; University of the Philippines, Los Baños, Philippines

**Keywords:** Copra expellers, Copra meal, Palm kernel expellers, Palm kernel meal, Rice bran

## Abstract

High fiber co-products from the copra and palm kernel industries are by-products of the production of coconut oil and palm kernel oil. The co-products include copra meal, copra expellers, palm kernel meal, and palm kernel expellers. All 4 ingredients are very high in fiber and the energy value is relatively low when fed to pigs. The protein concentration is between 14 and 22 % and the protein has a low biological value and a very high Arg:Lys ratio. Digestibility of most amino acids is less than in soybean meal but close to that in corn. However, the digestibility of Lys is sometimes low due to Maillard reactions that are initiated due to overheating during drying. Copra and palm kernel ingredients contain 0.5 to 0.6 % P. Most of the P in palm kernel meal and palm kernel expellers is bound to phytate, but in copra products less than one third of the P is bound to phytate. The digestibility of P is, therefore, greater in copra meal and copra expellers than in palm kernel ingredients. Inclusion of copra meal should be less than 15 % in diets fed to weanling pigs and less than 25 % in diets for growing-finishing pigs. Palm kernel meal may be included by 15 % in diets for weanling pigs and 25 % in diets for growing and finishing pigs.

Rice bran contains the pericarp and aleurone layers of brown rice that is removed before polished rice is produced. Rice bran contains approximately 25 % neutral detergent fiber and 25 to 30 % starch. Rice bran has a greater concentration of P than most other plant ingredients, but 75 to 90 % of the P is bound in phytate. Inclusion of microbial phytase in the diets is, therefore, necessary if rice bran is used. Rice bran may contain 15 to 24 % fat, but it may also have been defatted in which case the fat concentration is less than 5 %. Concentrations of digestible energy (DE) and metabolizable energy (ME) are slightly less in full fat rice bran than in corn, but defatted rice bran contains less than 75 % of the DE and ME in corn. The concentration of crude protein is 15 to 18 % in rice bran and the protein has a high biological value and most amino acids are well digested by pigs. Inclusion of rice bran in diets fed to pigs has yielded variable results and based on current research it is recommended that inclusion levels are less than 25 to 30 % in diets for growing-finishing pigs, and less than 20 % in diets for weanling pigs. However, there is a need for additional research to determine the inclusion rates that may be used for both full fat and defatted rice bran.

## Background

With the increased global production of livestock, dairy, and poultry, the demand for feed is also increasing and co-products from the tropical food industries are increasingly used in diets fed to pigs. These co-products include copra meal and copra expellers, palm kernel meal and palm kernel expellers, and rice bran. Global production of palm kernel and copra products is relatively modest compared with the production of soybean meal and canola meal [1] and availability of these ingredients is often geographically dependent. However, in some areas, these ingredients are the most abundant and least expensive sources of energy and amino acids (AA) that are available to the local swine industry [2] and it is, therefore, important that information about the nutritional value of each ingredient is available. It is also recognized that copra-, palm kernel-, and rice co-products have certain specific attributes including AA profile, fatty acid profile, and composition of fiber that are unique to these ingredients and special attention to these attributes is needed. The objective of this review, therefore, is to provide information about the composition and recommended use of copra- and palm kernel products and of defatted and full fat rice bran when fed to pigs. The review is primarily based on literature published in peer-reviewed journals and feed composition tables. Although attempts were made to mainly include data that have been published since 1990, on a few occasions, it was necessary to include older data because of a lack of newer data for some of these ingredients. With the exception of ingredient tables, non-peer-reviewed literature was not used because of the uncertainty of the quality of this information.

## Copra meal and copra expellers

The coconut palm (*Cocos nucifera*) is widely distributed throughout the tropics with major production in Indonesia, The Philippines, India, and in some African and Central American and South American countries. World production of copra meal and copra expellers is approximately 2,000,000 metric tons [1].

Copra meal is produced by expeller extracting or solvent extracting the oil from dried coconut kernels. Copra meal is sometimes referred to as coconut meal or coconut oil meal. Although its protein content is less than that of conventional ingredients commonly used as protein sources, copra meal represents the largest quantity of locally available feed protein in many tropical areas, such as countries in Central America, some African countries, and some countries in South East Asia. Variations in the nutrient composition of copra meal are mainly a function of the differences in residual oil concentration.

The residual oil in copra meal and copra expellers contains 50 to 70 % medium-chain, saturated fatty acids (lauric acid and myristic acid), which can lead to firmer carcass fat when high levels of copra meal are used in the diet [3, 4]. Thus, the deposition of lauric acid and myristic acid is three to five times greater in the backfat of pigs fed 30 % copra meal compared with pigs fed 10 % copra meal [3]. Quality problems such as rancidity and aflatoxin contamination may be an issue in copra meal, which may cause reduced feed intake and in some cases reduced feed efficiency of pigs [5]. Quality problems may be attributed to the high moisture content of copra during drying and storage [6].

Copra meal and copra expellers contain between 10 and 16 % crude fiber and approximately 47 % total dietary fiber [7]. Concentrations of β-mannans, galactomannans, arabinoxylogalactans, and cellulose are relatively high [8, 9] and the water binding capacity of copra meal is much greater than that of palm kernel meal or palm kernel expellers [7]. Water binding capacity is an estimate of the amount of water that a fiber can absorb and hold after an external force has been applied to it via centrifugation. High water binding capacity will usually result in reduced feed intake of animals because of swelling in the intestinal tract. The relatively high concentrations of fermentable fiber in copra- and palm kernel ingredients may result in increased needs for dietary Thr because dietary fiber increases the endogenous losses of AA, and therefore increases the loss of Thr [9]. Protein levels of copra meal and copra expellers typically range from 20 to 26 % (Table 1). The concentration of gross energy in copra meal is greater than in corn, but because of the high concentration of fiber in copra meal and copra expellers, concentrations of digestible energy (DE) and metabolizable energy (ME) are less than in corn [10, 11].

**Table 1:** Energy and nutrient composition and physical characteristics of copra and palm kernel ingredients (as-fed basis)^1^

		Ingredient			
Item^2^	Copra meal	Copra expellers	Palm kernel expellers	Palm kernel meal	Soybean meal
Dry matter, %	92.9	89.9	91.9	91.9	90.0
Bulk density, g/L	502.4	-	634.1	401.0	-
Water binding capacity, g/g	4.18	-	1.83	2.17	-
Gross energy, MJ/kg	18.6	18.0	18.8	17.8	17.8
DE, MJ/kg	14.4	15.8	12.1	11.3	15.2
ME, MJ/kg	13.6	15.1	11.7	10.6	13.8
NE, MJ/kg	7.3	-	8.1	6.9	8.7
Crude protein, %	22.0	20.2	14.3	13.6	47.7
Acid hydrolyzed ether extract, %	1.9	7.1	6.9	1.3	2.9
Neutral detergent fiber, %	54.8	54.4	70.6	77.9	8.2
Acid detergent fiber, %	26.9	29.6	43.0	49.4	5.3
Insoluble dietary fiber, %	41.4	-	60.9	68.7	16.7
Soluble dietary fiber, %	5.5	-	2.6	2.2	-
Total dietary fiber, %	46.9	-	63.5	70.9	-

^1^References [6, 9, 10, 15]

Copra meal and copra expellers contain between 0.50 and 0.58 % total P [10, 12, 13], but less than one third of the P is bound to phytate. The standardized total tract digestibility (STTD) of P, therefore, is relatively high in copra meal and copra expellers (Table 2; [12, 13]). However, if microbial phytase is included in the diets, the STTD of P will increase [13].

**Table 2:** Concentrations of minerals, phytate, and apparent total tract digestibility (ATTD) and standardized total tract digestibility (STTD) of phosphorus in copra and palm kernel ingredients (as-fed basis)^1^

Item	Copra meal	Copra expellers	Palm kernel meal	Palm kernel expellers	Soybean meal
Ca, %	0.04	0.11	0.20	0.25	0.33
Cl, %	0.37	-	-	0.15	0.05
K, %	1.83	1.75	-	0.64	2.11
Mg, %	0.31	0.23	-	0.29	0.28
Na, %	0.04	-	-	0.03	0.05
P, %	0.52	0.53	0.54	0.52	0.67
S, %	0.31	-	-	0.22	0.4
Cu, ppm	25	40	-	21	16.5
Fe, ppm	486	-	-	534	190.5
Mn, ppm	69	75	-	131	36.7
Zn, ppm	49	53	-	32	47.9
Phytate, %	0.79	0.78	1.12	1.29	1.55
Phytate P, %	0.22	0.22	0.32	0.35	0.44
Non-phytate P, %	0.30	0.31	0.22	0.16	0.23
P digestibility without phytase					
ATTD, %	60.8	46.0	48.9	30.0	41.1
STTD, %	70.6	56.5	57.9	39.8	49.6
P digestibility with phytase					
ATTD, %	80.8	-	64.1	58.2	72.2
STTD, %	90.3	-	73.5	68.1	81.1

^1^References [2, 9, 11, 12, 46]

The quality of the protein in copra meal is less than that of soybean meal and palm kernel products with Lys only being 1.91 % of total crude protein (CP) and total indispensable AA being 33.92 % of total CP. However, one specific characteristic of copra protein is that it is high in Arg and Arg is almost 10 % of total CP and the Arg:Lys ratio is almost 5:1 (Table 3).

**Table 3:** Amino acid (AA) composition in copra and palm kernel ingredients (as-fed basis)^1^

Item	Copra meal		Copra expellers		Palm kernel meal		Palm kernel expellers		Soybean meal	
	%	% of CP	%	% of CP	%	% of CP	%	% of CP	%	% of CP
Crude protein	22.00	-	20.20	-	13.60	-	14.30	-	47.73	-
Indispensable AA										
Arg	2.08	9.45	1.70	8.42	1.36	10.00	1.52	10.63	3.45	7.23
His	0.35	1.59	0.29	1.44	0.17	1.25	0.20	1.40	1.28	2.68
Ile	0.66	3.00	0.06	0.30	0.41	3.01	0.47	3.29	2.14	4.48
Leu	1.20	5.45	1.19	5.89	0.71	5.22	0.82	5.73	3.62	7.58
Lys	0.42	1.91	0.39	1.93	0.36	2.65	0.36	2.52	2.96	6.20
Met	0.27	1.23	0.24	1.19	0.22	1.62	0.25	1.75	0.66	1.38
Phe	0.79	3.59	0.79	3.91	0.47	3.46	0.53	3.71	2.40	5.03
Thr	0.55	2.50	0.57	2.82	0.33	2.43	0.37	2.59	1.86	3.90
Trp	0.15	0.68	0.15	0.74	0.05	0.37	0.12	0.84	0.66	1.38
Val	0.97	4.41	0.91	4.50	0.57	4.19	0.65	4.55	2.23	4.67
Total	7.44	33.81	6.29	31.14	4.65	34.20	5.29	37.01	21.26	44.53
Dispensable AA										
Ala	0.85	3.86	0.79	3.91	0.46	3.38	0.53	3.71	2.06	4.32
Asp	1.50	6.82	1.49	7.38	0.89	6.54	0.99	6.92	5.41	11.33
Cys	0.28	1.27	0.26	1.29	0.17	1.25	0.17	1.19	0.70	1.47
Glu	3.34	15.18	3.43	16.98	2.02	14.85	2.29	16.01	8.54	17.89
Gly	0.82	3.73	0.82	4.06	0.53	3.90	0.58	4.06	1.99	4.17
Pro	0.60	2.73	0.63	3.12	0.36	2.65	0.40	2.80	2.53	5.30
Ser	0.71	3.23	0.77	3.81	0.44	3.24	0.50	3.50	2.36	4.94
Tyr	0.41	1.86	0.54	2.67	0.29	2.13	0.29	2.03	1.59	3.33
Total	8.51	38.68	8.73	43.22	5.16	37.94	5.75	40.22	25.18	52.75
All AA										
Arg:Lys, %	4.95	-	4.36	-	3.78	-	4.22	-	1.17	-

^1^References [9, 10, 15]

The standardized ileal digestibility (SID) of AA in copra meal and copra expellers fed to pigs ranges between 43 and 81 % [11, 14–16]. The SID of Lys in copra meal is also variable, ranging from 51 [15, 17] to 73 % [10], but the SID of all other indispensable AA is greater than that of Lys indicating that the sources of copra meal used in these experiments may have been heat damaged because heat damage will reduce the digestibility of Lys more than that of other AA [18–20]. The SID of Lys in copra expellers was reported at only 40 % [16], which was much less than for other indispensable AA indicating that this source was also heat damaged. The differences in AA digestibility among experiments may also be due to differences in nutrient composition, drying procedures, oil extraction procedures, and the degree and duration of heat processing that is used during oil extraction [21]. Overall, the SID of protein and indispensable AA in copra expellers is less than in soybean meal, but similar to those in palm kernel meal (Table 4; [11]).

**Table 4:** Standardized ileal digestibility (%) of amino acids (AA) in copra and palm kernel products and in soybean meal^1^

Item	Copra meal	Copra expellers	Palm kernel meal	Palm kernel expellers	Soybean meal
Crude protein	79.9	67.6	71.3	81.8	87.0
Indispensable AA					
Arg	91.2	90.0	88.3	90.4	94.0
His	82.5	73.2	80.8	83.6	90.0
Ile	81.6	76.7	80.4	83.5	89.0
Leu	81.6	78.5	79.7	82.4	88.0
Lys	72.8	40.3	71.1	76.5	89.0
Met	85.5	82.1	82.2	85.0	90.0
Phe	84.5	81.4	82.2	84.6	88.0
Thr	76.7	64.4	73.9	77.2	85.0
Trp	88.4	66.3	87.5	89.4	91.0
Val	79.0	77.8	77.2	81.0	87.0
Mean	82.6	73.1	80.3	83.4	89.1
Dispensable AA					
Ala	78.2	79.0	72.5	79.0	85.0
Asp	78.9	66.5	75.8	77.5	87.0
Cys	68.0	53.1	71.7	76.4	84.0
Glu	79.9	67.3	81.2	82.0	89.0
Gly	76.2	60.6	65.1	77.9	84.0
Pro	128.8	125.0	54.9	121.5	113.0
Ser	82.0	70.5	80.0	83.7	89.0
Tyr	82.8	58.0	80.1	82.7	88.0
Mean	83.7	72.5	75.2	83.7	89.9
Mean all AA	83.2	72.8	77.6	83.5	89.5

^1^Refereces [9, 10, 15]

Copra meal and copra expellers may be included in diets fed to growing and finishing pigs by up to 30 % without affecting growth performance [22], but negative effects of increasing levels of copra meal in the diet have been reported [4, 14, 23]. However, Thorne et al. [3] demonstrated that copra meal can be used by up to 50 % in growing-finishing diets if diets are supplemented with synthetic AA or proteins with higher quality. Results with copra meal have been improved if diets either were semi-purified diets or if they were formulated based on digestible AA rather than based on crude protein [3].

In diets fed to weanling pigs from 2 wk post-weaning, performance was linearly reduced if copra meal was included in the diet and pigs fed diets containing 15 % copra meal gained approximately 1 kg less over a 3-wk period than pigs fed a control diet without copra meal [7]. This result was obtained even though diets were balanced for digestible AA and ME. It is possible that it is the high fiber concentration and the high water binding capacity of the fiber in copra meal that resulted in the pigs eating less and therefore gaining less weight because of the increased gut fill that is associated with consuming diets with high water binding capacity. However, gain to feed ratio was also reduced over the 3-wk feeding period if copra meal was used. It is, therefore, recommended that less than 15 % copra meal is used in diets fed to weanling pigs.

## Palm kernel meal and palm kernel expellers

Global production of palm kernel meal and palm kernel expellers has increased from approximately 5 million metric tons in 2005 to almost 7 million metric tons in 2012 [1]. The reason for this increase is the increased demand for palm oil, which is often used in the biodiesel industry. Produced mainly in Southeast Asia and Africa, the oil palm fruit (*Elaeis guineensis*) yields palm oil extracted from the fleshy, outer mesocarp that surrounds the nut and palm kernel oil extracted from the kernel within the inner, hard shelled nut [24]. Prior to oil extraction, the outer shell of the kernel is cracked open, separated, and subjected to steam conditioning. Mechanical extraction by screw pressing is the most common process in oil extraction from palm kernels, which results in production of palm kernel expellers. However, sometimes oil is removed via solvent extraction, and the resultant co-product is called palm kernel meal.

The nutrient concentration of palm kernel meal and palm kernel expellers depends on the method of oil extraction, the species of the palm nut, and the amount of shell remaining in the meal [25]. Palm kernel expellers have a residual oil concentration of 6 to 8 %, whereas solvent-extracted meals contain 1 to 2 % residual oil (Table 1; [26, 27]). The concentration of crude fiber in palm kernel meal ranges between 7 and 20 % [28], depending on the amount of shells and fruit removed from the palm kernel. More than 81 % of the total carbohydrates in palm kernel meal are in the form of non-starch polysaccharides [29], mainly as β-(1,4)-D-mannans [30, 31]. Palm kernel meal also contains high amounts of lignin, which may be a result of contamination of nut shells [32], which contributes to its grittiness and fibrous texture. However, water binding capacity in palm kernel meal and palm kernel expellers is less than in copra meal [7]. Because of the high concentration of insoluble dietary fiber, the energy in palm kernel meal and palm kernel expellers is poorly digested by pigs and concentrations of DE and ME in palm kernel meal and palm kernel expellers is less than 75 % of that in soybean meal and corn (Table 1; [10, 11]). However, energy digestibility in diets containing palm kernel expellers may be increased by 2 to 3 percentage units if beta-mannanase is added to the diet [32] because beta-mannanase may help digesting some of the D-mannans in palm kernel expellers.

The concentration of P in palm kernel meal and palm kernel expellers is between 0.5 and 0.65 % [10, 12, 13]. However, between 60 and 75 % of total P is bound to phytate and the STTD of P in palm kernel meal and palm kernel expellers is, therefore, between 35 and 50 % (Table 2; [10, 12, 13]). Because of the relatively high concentration of phytate in palm kernel products, the STTD of P can be increased to between 60 and 75 % if microbial phytase is added to the diets[13]. As a consequence, the supply of digestible P from palm kernel meal and palm kernel expellers is similar to that of soybean meal if microbial phytase is added to the diet [13].

Relative to other oilseed meals, palm kernel meal has the lowest protein concentration ranging from 14 to 21 % [11, 26]. Palm kernel protein has a low concentration of Trp and a relatively high concentration of Arg, which is approximately 10 % of the CP (Table 3; [11, 33]). However, the Arg:Lys ratio is around 4:1 (Table 3) and as is the case with copra co-products, the supply of Arg is much greater than if other feed ingredients are used. The high concentration of Arg may suppress the digestibility of Lys because Arg and Lys compete for the same transporter in the enterocytes [34, 35]. However, making sure that diets are sufficient in digestible Lys may minimize the negative effect of high concentration of Arg. In general, the standardized ileal digestibility of AA in both palm kernel meal and palm kernel expellers is less than in soybean meal, but not different from copra meal (Table 4; [11, 36, 37]).

Palm kernel meal and palm kernel expellers are not always well-accepted by pigs [38, 39] and if included by more than 20 % in the diet, palm kernel meal negatively affects growth performance and carcass quality of growing finishing pigs [40, 41]. It is, however, possible that if diets are formulated to be equal in standardized ileal digestible indispensable AA, pigs will be able to perform better on diets containing palm kernel meal and palm kernel expellers. Finishing pigs have greater tolerance for palm kernel meal than nursery pigs [28]. In experiments with weanling pigs, it was observed that if diets are formulated to contain similar concentrations of digestible AA and ME, feed conversion rates may be maintained if up to 15 % palm kernel meal or palm kernel expellers are included in the diets [7]. However, average daily gain may be slightly reduced if palm kernel products are used, which may be a result of reduced bulk density of the diet and increased water binding capacity [7].

## Full fat rice bran and defatted rice bran

The global production of rice (*Oryza sativa*) exceeds 700 million metric tons per year and rice is the most produced cereal grain in the world after maize and wheat [42]. Rice is produced primarily for human consumption and is the main carbohydrate source in human diets in many countries in the world. The largest rice producing countries are China and India followed by Indonesia, Vietnam, and Thailand [42]. Annual production of rice in the United States is around 9 million metric tons, but the United States is the 5^th^ largest exporter of rice after Thailand, India, Vietnam, and Pakistan.

The main objective of producing rice is to produce polished white rice that is used for human consumption. However, paddy rice contains approximately 20 % hulls that mainly consist of lignin and silica, and therefore, has very low nutritional value [43]. As a consequence, rice has to be de-hulled before consumption. Removal of the hulls results in production of brown rice that contains the bran layers, the germ, and the endosperm. Further processing is needed to remove the bran layers and endosperm and this results in production of rice bran, which may be used for animal feeding. After the bran has been removed, rice goes through several polishing steps before the final product, polished rice, is produced [44]. On a quantitative basis, rice bran is approximately 10 % of the total weight of paddy rice, which means that approximately 70 million metric tons of rice bran is produced annually and is available for animal feeding. There are other co-products produced from rice including brewers rice and rice mill feed, but these products are produced in much smaller quantities.

Rice bran includes the pericarp, the aleurone, and the subaleurone layers of rice, but depending on the type of milling, fractions of the endosperm may make up 20 to 25 % of the bran product [45]. Rice bran, therefore, may contain up to 30 % starch [10, 46]. The concentration of ether extract in rice bran varies between 14 and 24 % depending on the variety of rice that was grown and the type of milling used [10, 46, 47]. However, because of the high concentration of lipase in rice bran, the fat may quickly peroxidize and become rancid [45, 48]. As a consequence, rice bran needs to be stabilized by use of heat treatment such as extrusion to deactivate the lipase and thus reduce the risk of oxidation [49]. Alternatively, the fat may be removed from rice bran using solvent extraction to produce defatted rice bran with a concentration of fat of 2 to 4 %. Therefore, both full fat rice bran and defatted rice bran are available for animal feeding.

Full fat rice bran contains 20 to 30 % neutral detergent fiber and the concentration of CP is approximately 15 % [10, 46, 47]. Values for DE in full fat rice bran have been reported between 3,000 and 3,100 kcal per kg and values for ME are approximately 100 kcal less than the DE values (Table 5; [10, 46]). Concentrations of neutral detergent fiber and CP in defatted rice bran are 10 to 15 % greater than in full fat rice bran because removal of the fat concentrates other nutrients in the bran. However, DE and ME values in defatted rice bran are much less than in full fat rice bran and values between 2,100 and 2,200 kcal per kg have been reported [10, 46].

**Table 5:** Energy and nutrient composition of full fat rice bran and defatted rice bran (as-fed basis)^1^

	Ingredient	
Item	Full fat rice bran	Defatted rice bran
Dry matter, %	91.60	91.35
Ash, %	14.80	11.51
Gross energy, MJ/kg	19.98	16.98
Digestible energy, MJ/kg	12.98	9.21
Metabolizable energy, MJ/kg	12.55	8.71
Net energy, MJ/kg	9.54	6.50
Crude protein, %	15.11	17.30
Acid hydrolyzed ether extract, %	13.77	3.52
Starch, %	27.00	26.25
Neutral detergent fiber, %	26.28	23.56
Acid detergent fiber, %	11.87	11.31
Minerals		
Ca, %	0.22	0.17
Cl, %	0.08	0.10
K, %	1.45	1.11
Mg, %	0.72	0.81
Na, %	0.04	0.02
P, %	2.16	1.89
Phytate-P, %	1.74	1.61
Non-phytate P, %	0.42	0.28
S, %	0.18	0.15
Cu, ppm	8.00	14.00
Fe, ppm	113.00	268.00
Mn, ppm	219.50	267.00
Zn, ppm	45.70	73.00
ATTD^2^ of P without phytase, %	24.00	12.00
ATTD of P with phytase, %	62.00	-
STTD^3^ of P witout phytase, %	36.50	28.00
STTD of P with phytase, %	64.00	-

^1^References [9, 42, 46, 59]

^2^ATTD, apparent total tract digestibility

^3^STTD, standardized total tract digestibility

The concentration of P is greater in rice bran than in most other plant ingredients and values between 1.6 and 2.2 % have been reported [10, 46, 50]. Between 70 and 90% of the P is bound in phytate, and the STTD of P in rice bran, therefore, is relatively low (Table 5; [50, 51]). However, addition of 1,000 units/kg of microbial phytase will increase the STTD of P in rice bran by 15 to 50 % [51].

The biological value of rice protein is high and the standardized ileal digestibility of most AA in polished rice is greater than in most other cereal grains except wheat [52]. The protein in rice bran also has a relatively high concentration of Lys, Met, Trp, and Thr (Table 6). However, the SID of AA in both full fat and defatted rice bran is considerably less than in polished rice and for most indispensable AA, values between 70 and 85 % have been reported (Table 6; [10, 47]).

**Table 6:** Amino acid (AA) composition and standardized ileal digestibility (SID) of AA in full fat rice bran and defatted rice bran (as-fed basis)^1^

Item	Full fat rice bran			Defatted rice bran		
	%	% of CP	SID, %	%	% of CP	SID, %
Crude protein	14.80	-	-	16.27	-	-
Indispensable AA						
Arg	1.15	7.79	93.0	1.31	8.07	90.5
His	0.38	2.59	87.8	0.45	2.74	82.7
Ile	0.49	3.34	83.2	0.68	3.54	78.4
Leu	0.98	6.66	82.9	1.15	7.06	77.7
Lys	0.64	4.35	85.8	0.75	4.59	82.3
Met	0.29	1.95	87.3	0.33	2.05	78.7
Phe	0.62	4.18	81.1	0.72	4.40	78.0
Thr	0.52	3.51	80.6	0.62	3.79	77.0
Trp	0.18	1.19	83.0	0.21	1.27	79.7
Val	0.75	5.04	83.9	0.87	5.35	79.0
Total	6.00	40.54	-	7.09	43.58	-

^1^Reference [9, 46, 60]

There are relatively few reports on effects of including rice bran in diets fed to weanling, growing, or finishing pigs. However, inclusion of 10 % rice bran in diets fed to weanling pigs improved feed conversion rate by almost 10 % because of increased colonic concentrations of bifidobacteria [53]. A balanced microbial community with a large presence of the beneficial bacteria is critical for weanling pigs to maintain their intestinal health. The prebiotic effect of rice bran was likely related to arabinoxylan oligosaccharides in this ingredient [54, 55]. However, it is not known what the maximum inclusion rate is. For growing and finishing pigs, reduced growth performance has been reported for inclusion of 30 % full fat rice bran [56]. Inclusion of 10 % full fat rice bran in diets fed to growing pigs had no influence on the growth performance compared with pigs fed a corn-soybean meal control diet [50]. In finishing diets, inclusion of 20 % full fat rice bran improved performance compared with pigs fed defatted rice bran [57], and it has been suggested that the maximum inclusion rate of defatted rice bran in diets fed to growing-finishing pigs is 20 % [58]. It is, however, possible, that the reduced performance of pigs fed the defatted rice bran simply is an effect of the reduced metabolizable energy in the defatted rice bran. If that is the case then it is expected that the reduction in growth performance observed for pigs fed defatted rice bran can be avoided if diets are formulated to be isocaloric. However, to our knowledge, research to test this hypothesis has not been reported.

## Abbreviations

AA: Amino acids
ATTD: Apparent total tract digestibility
CP: Crude protein
DM: Dry matter
DE: Digestible energy
ME: Metabolizable energy
SID: Standardized ileal digestibility
STTD: Standardized total tract digestibility
